# Zygotic Splicing Activation of the Transcriptome is a Crucial Aspect of Maternal‐to‐Zygotic Transition and Required for the Conversion from Totipotency to Pluripotency

**DOI:** 10.1002/advs.202308496

**Published:** 2024-02-02

**Authors:** Hua Zhang, Yang Wang, Zhe‐Wei Hu, Yun‐Wen Wu, Nuo Chen, Yi‐Min Zhu, Yuan‐Song Yu, Heng‐Yu Fan, Hua‐Nan Wang

**Affiliations:** ^1^ MOA Key Laboratory of Animal Virology Center for Veterinary Sciences Zhejiang University Hangzhou 310058 China; ^2^ Department of Veterinary Medicine College of Animal Sciences Zhejiang University Hangzhou 310058 China; ^3^ MOE Key Laboratory for Biosystems Homeostasis and Protection and Innovation Center for Cell Signaling Network Life Sciences Institute Zhejiang University Hangzhou 310058 China; ^4^ Department of Reproductive Endocrinology Women's Hospital School of Medicine Zhejiang University Hangzhou Zhejiang 310002 China; ^5^ Savaid Stomatology School Hangzhou Medical College Hangzhou 310053 China; ^6^ Assisted Reproduction Unit Department of Obstetrics and Gynecology Sir Run Run Shaw Hospital School of Medicine Zhejiang University Hangzhou 310016 China; ^7^ Center for Biomedical Research Shaoxing Institute Zhejiang University Shaoxing 312000 China

**Keywords:** alternative splicing, early embryo, RNA processing, splicing factors, totipotency and pluripotency, zygotic genome activation

## Abstract

During maternal‐to‐zygotic transition (MZT) in the embryo, mRNA undergoes complex post‐transcriptional regulatory processes. However, it is unclear whether and how alternative splicing plays a functional role in MZT. By analyzing transcriptome changes in mouse and human early embryos, dynamic changes in alternative splicing during MZT are observed and a previously unnoticed process of zygotic splicing activation (ZSA) following embryonic transcriptional activation is described. As the underlying mechanism of RNA splicing, splicing factors undergo dramatic maternal‐to‐zygotic conversion. This conversion relies on the key maternal factors BTG4 and PABPN1L and is zygotic‐transcription‐dependent. CDK11‐dependent phosphorylation of the key splicing factor, SF3B1, and its aggregation with SRSF2 in the subnuclear domains of 2‐cell embryos are prerequisites for ZSA. Isoforms generated by erroneous splicing, such as full‐length *Dppa4*, hinder normal embryonic development. Moreover, alternative splicing regulates the conversion of early embryonic blastomeres from totipotency to pluripotency, thereby affecting embryonic lineage differentiation. ZSA is an essential post‐transcriptional process of MZT and has physiological significance in generating new life. In addition to transcriptional activation, appropriate expression of transcript isoforms is also necessary for preimplantation embryonic development.

## Graphical abstract

This study reports on the dynamic changes in alternative splicing during the maternal‐to‐zygotic transition (MZT) and describes the previously unnoticed process of zygotic splicing activation (ZSA). Zygotic splicing activation depends on key maternal factors BTG4 and PABPN1L and is zygotic transcription‐dependent. Zygotic alternative splicing also regulates the conversion of early embryonic blastomeres from totipotency to pluripotency.

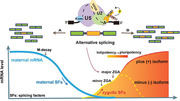

## 1 Introduction

During mammalian embryonic development, the transition from the maternal to the zygotic transcriptome occurs in all embryos. This maternal‐to‐zygotic transition (MZT) process can be subdivided into two coupling stages: the degradation of maternal transcripts and the initiation of zygotic transcription.^[^ ^1^ ^]^ The degradation of maternal mRNA is the core event in MZT and is a prerequisite for zygotic genome activation (ZGA).^[^ ^2^ ^]^ Recent studies have indicated that some maternal factors, such as BTG4 and PABPN1L, mediate maternal mRNA decay in mouse oocytes and zygotes by recruiting the CCR4‐NOT deadenylase complex.^[^ ^3^, ^4^, ^5^, ^6^, ^7^, ^8^ ^]^ ZGA occurs in two obvious waves of increasing degrees: minor ZGA and major ZGA. In mice, minor ZGA occurs at the zygote stage, and the global activation of zygotic genes at the mid‐to‐late 2‐cell stage is known as major ZGA to distinguish it from minor ZGA.^[^ ^9^, ^10^ ^]^ However, in humans, the major wave of ZGA occurs at the 4–8‐cell stage.^[^ ^11^ ^]^ In recent years, based on RNA sequencing and other related technologies, we have further elucidated the activation of the zygotic genome in early embryos and confirmed that the normal operation of ZGA is essential for early embryo development. However, these studies have mainly focused on the transcriptional expression level changes of protein‐coding genes, and research on post‐transcriptional regulatory changes in some RNAs is insufficient.

Alternative splicing is an important post‐transcriptional RNA processing mechanism that results in the formation of different mRNA isomers by selecting different splicing site combinations of the precursor RNA (pre‐RNA). This process can produce different protein isomers and it plays an important role in maintaining cell homeostasis and regulating cell differentiation and development,^[^ ^12^, ^13^, ^14^ ^]^ thus significantly expanding the regulatory capacity of the genome.^[^ ^15^, ^16^ ^]^ The regulation of alternative splicing is as important as the regulation of transcription in multicellular eukaryotes.^[^ ^16^ ^]^ However, not all alternatively spliced transcripts produce functional proteins and some mRNA isoforms harbor premature termination codons. These transcripts are predicted to be degraded via nonsense‐mediated mRNA decay (NMD) pathways, thereby regulating gene expression.^[^ ^17^ ^]^ The regulation of alternative splicing is often described in terms of splicing factors that guide or block spliceosome assembly at specific splice sites.^[^ ^18^ ^]^ Spliceosomes are mainly composed of five small nuclear ribonucleoproteins (U1, U2, U4/U6, and U5) and several cofactors.^[^ ^19^, ^20^ ^]^ During the spliceosome assembly process, there are 10 major functional stages, referred to as the E, A, pre‐B, B, B^act,^ B*, C, C*, P, and intron‐lariat spliceosomal complexes.^[^ ^21^, ^22^, ^23^ ^]^ SF3B1 and SRSF2 (SC35) are key splicing factors, and their mutations lead to disrupt splicing and are involved in cancer pathogenesis.^[^ ^24^ ^]^

Pladienolide B (PlaB) is a small‐molecule drug that selectively binds the splicing factor SF3B1, preventing it from recognizing branch sites of introns and thus interfering with normal splicing.^[^ ^25^, ^26^, ^27^ ^]^ A recent study used PlaB to achieve a stable in vitro culture of totipotent embryonic stem cells, known as totipotent blastomere‐like cells (TBLCs), that are comparable at the molecular level to 2‐ and 4‐cell blastomeres.^[^ ^28^ ^]^ SF3B1 requires phosphorylation during spliceosome activation. A recent study reported that CDK11 is the kinase directly responsible for SF3B1 phosphorylation. The use of OTS964, a selective inhibitor of CDK11, successfully suppresses the phosphorylation of SF3B1.^[^ ^29^ ^]^ Alternative splicing and its regulatory processes are believed to be closely associated with cancer characteristics, and numerous splicing factors, such as SF3B1, have been demonstrated to be involved in tumorigenesis.^[^ ^20^, ^30^, ^31^, ^32^ ^]^ However, the changes in these splicing factors during MZT and the regulatory role of alternative splicing in embryonic development have not been well elucidated.

It has been observed that RNA splicing activity is not active in the zygote stage, but the specific role of alternative splicing in subsequent embryonic development has not been fully elucidated.^[^ ^33^, ^34^, ^35^, ^36^ ^]^ Evidence suggests that alternative splicing plays a crucial role in regulating maternal transcripts in oocytes.^[^ ^37^, ^38^ ^]^ Defects in maternal splicing factors can impair oocyte maturation and affect female fertility in mice.^[^ ^39^, ^40^ ^]^ However, further studies are required to clarify the relationship between pre‐RNA splicing and early embryonic development.

Surprisingly, alternative splicing has been found to be closely related to the totipotent/pluripotent transformation of embryonic stem cells.^[^ ^28^ ^]^ Previous studies have shown that alternative splicing begins at the 2‐cell stage of preimplantation mouse embryos, which is also a major stage in the transition between the totipotency and pluripotency of embryos.^[^ ^33^, ^34^, ^35^, ^36^, ^41^ ^]^ Several studies have investigated the effects of alternative splicing on oocyte maturation.^[^ ^37^, ^39^, ^40^ ^]^ However, the acquisition of RNA splicing capabilities during early embryonic development has not been sufficiently explored. The interplay between alternative splicing and ZGA, as well as the physiological importance of alternative splicing in oocytes and early embryonic development, require further elucidation. The present study demonstrates significant changes in alternative splicing events (ASEs) during early embryonic development, coinciding with major ZGA. We investigated the physiological significance and regulatory mechanisms of alternative splicing during early embryonic development.

## 2 Results

### 2.1 Alternative Splicing Activation Occurs at the ZGA Stage

**Figure 1:**
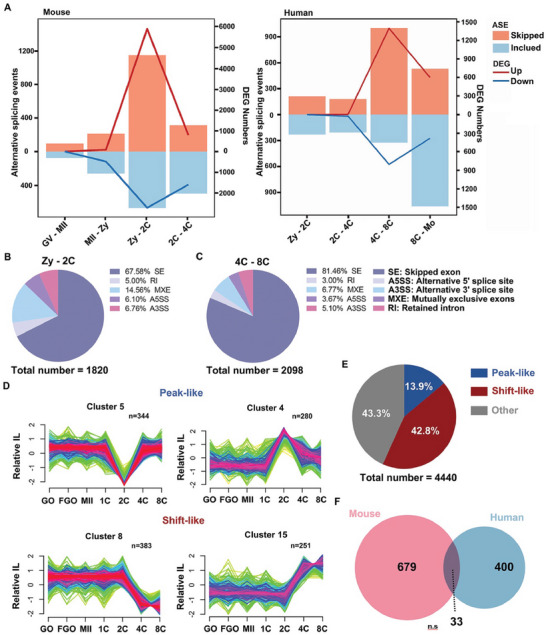
Dynamic changes in alternative splicing in human and mouse early embryos. A) The alternative splicing event (ASE, FDR < 0.05 and |ILD| > 0.1) and differentially expressed genes (DEGs, FDR < 0.05 and FPKM |Log2 fold change| > 2 or < 0.5) number of mouse oocytes and early embryos in different stages. FDR, false discovery rate; ILD, IncLevelDifference; FPKM, fragments per kilobase of transcript per million mapped reads. B) The ratio of different ASEs in mouse embryos at the 2‐cell stage. C) The ratio of different ASEs in human embryos at the 4‐ to 8‐cell stage. D) Representative examples of two major mouse IncLevel (IL) cluster types (peak‐like and shift‐like) from Mfuzz. E) The ratio of two major cluster types in all stages. The white numbers show the percentages of peak‐like, shift‐like, and other types. F) Venn diagrams showing the overlap of genes with ASEs from mice and humans (FDR > 0.05, (|ILD| > 0.1). n.s.: non‐significant. GV, germinal vesicle oocyte; MII, MII oocyte; Zy, zygote; 2C, 2‐cell; 4C, 4‐cell; 8C, 8‐cell; and Mo, morula.

To investigate the dynamics of alternative splicing during oocyte and early embryo development, available RNA sequencing (RNA‐seq) datasets were used for ASE analysis.^[^ ^42^ ^]^ We used the exon inclusion level (IncLevel) to represent the proportion of different isoforms generated by alternative splicing, which ranged from 0 to 1. Furthermore, the IncLevelDifference (ILD) was used to represent the variation in ILD between the two sets of samples (Figure S1, Supporting Information). During mouse oocyte to early embryonic development, the number of ASEs is low at the germinal vesicle (GV) and metaphase II (MII) stages of the oocyte, whereas the number of ASEs increases significantly from the zygote to the 2‐cell stage, as well as at the 2–4‐cell stage, with most ASEs occurring at the 2‐cell stage (**Figure** **1**A; Figure S2A, Supporting Information). An analysis of early human embryonic RNA‐seq data revealed that human embryos undergo a large increase in the number of ASEs during the 4–8‐cell stage. This is precisely the time point of ZGA in early mouse and human embryos, respectively (Figure 1A; Figure S2B, Supporting Information). The ASEs were validated by reverse transcription polymerase chain reaction (RT‐PCR) using RNA from independent pools of embryos (Figure S2C, Supporting Information).

Common ASEs include intron retention (IR), skipped exon (SE), mutually exclusive exon (MXE), and alternative 5′ and 3′ splice sites (A5SS and A3SS). A large number of ASEs occur in the zygote to 2‐cell stage of early mouse embryos and the 4–8‐cell stage of early human embryos. We further distinguished the types of ASEs between the two groups of stages and found that they were mainly SEs, which occurred at proportions of 67.58% and 81.46% in mouse and human embryos, respectively. There was no significant difference in the proportion of ASEs at the different stages (Figure 1B,C; Figure S2D, Supporting Information). Based on these results, we found that there are dynamic changes in ASEs during early embryonic development in mice and humans and that the ASEs are predominantly SEs.

Therefore, the IncLevel values of each skipped exon event were further categorized according to changes in the IncLevel at different stages of early embryonic development. 16 and 14 exon clusters were identified in mice and humans (Figures S3 and S4 and Tables S1 and S2, Supporting Information). The mouse exon‐skipping clusters were classified according to their variation characteristics, and the following two types of clusters with obvious variation characteristics were found: peak‐like (the skipped or included exon occurred at a certain stage and then returned to normal levels in other stages) and shift‐like (the skipped or included exon transition occurred at a certain stage and did not return to the initial level). The variation nodes were in the range of 2–4‐cell stage (Figure 1D). The overall percentages of these two types of clusters were higher than those of the other clusters (Figure 1E). Cluster classification further describes the dynamic changes of ASEs, indicating that the alternative splicing processes of different genes have different regulatory patterns at the ZGA stage. Similar patterns were observed during early human embryonic development (Figure S4, Supporting Information). However, the overlap of the genes undergoing alternative splicing occurring in mouse zygote to 2‐cell and human 4–8‐cell was found to be low, indicating that the regulated genes of alternative splicing are species‐specific (Figure 1F). Taken together, these results suggest that zygotic splicing activation (ZSA) occurs during early mammalian embryonic development along with ZGA. However, the regulated genes showed interspecies specificity.

### 2.2 The Occurrence of ZSA Depends on the Normal Conduct of the MZT Process

**Figure 2:**
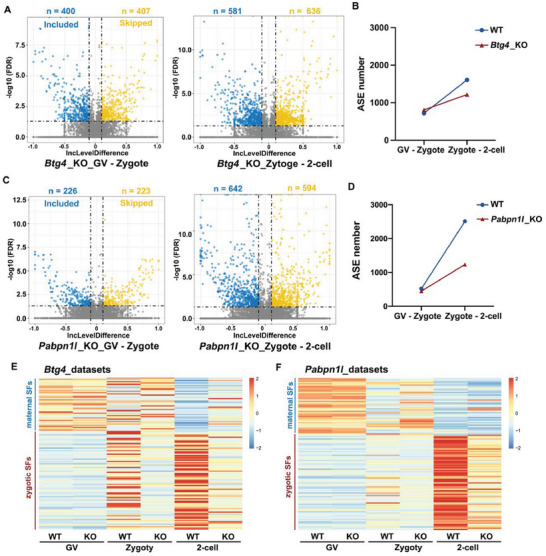
Failure of maternal‐to‐zygotic transition affects the activation of alternative splicing. A) The ASEs at different stages in *Btg4*‐knockout (KO) oocytes and embryos are shown with a volcano map. B) The total number of ASEs in *Btg4*‐wild‐type (WT) and *Btg4*‐KO oocytes at different stages. C) The ASEs at different stages in *Pabpn1l*‐KO oocytes and embryos are shown with a volcano map. D) The total number of ASEs in *Pabpn1l*‐WT and *Pabpn1l*‐KO oocytes at different stages. E,F) Heatmap showing the expression levels of different splicing factors in *Btg4*‐WT*, Btg4*‐KO, *Pabpn1l* ‐WT, and *Pabpn1l*‐KO oocytes and embryos.

As ZSA occurs along with the ZGA process, the connection between alternative splicing and MZT processes was further analyzed. *Btg4* and *Pabpn1l* are known to be key factors in M‐decay, and their knockout in oocytes leads to the arrest of embryonic development in the zygote up to 2‐cell embryo stage. However, the effect of maternal factors on ZSA remains unclear. Therefore, we analyzed ASEs using previously published RNA‐seq data from *Btg4‐* and *Pabpn1l*‐knockout mice. In the *Btg4‐*knockout mice dataset, the number of ASEs in zygotes to 2‐cell embryo stage increased compared to the number in the GV‐zygote stage (**Figure** **2**A), but the number of ASEs in zygotes to 2‐cell stage significantly decreased after *Btg4* knockout (Figure 2B). The data for *Pabpn1l*‐knockout embryos showed the same trend (Figure 2C,D). To determine the reasons for the impairment of ZSA due to maternal factor deletion, we analyzed changes in the transcript expression levels of splicing factors in the two groups of knockout mice.

Splicing factors are a class of proteins involved in intron removal and exon splicing of pre‐RNAs. We derived a complete list of splicing factors from published articles and “RNA splicing”‐ or “spliceosome”‐associated Gene Ontology (GO) terms from the Mouse Genome Informatics (MGI) database.^[^ ^35^, ^43^, ^44^, ^45^ ^]^ We separated 46 maternal splicing factors (more than a two‐fold decrease in the GV stage compared to the 2‐cell stage) and 81 zygotic splicing factors (more than a two‐fold increase in the 2‐cell stage compared to the GV stage) based on the changes in fragments per kilobase of transcript per million mapped reads (FPKM) values of splicing factors between GV oocytes and 2‐cell stage in both datasets. These two types of splicing factors exhibited significant dynamic trends during the MZT process. However, when the maternal factors BTG4 or PABPN1L were deleted, the heat map showed that the maternal splicing factors appeared to accumulate in the zygote and 2‐cell stages and could not be degraded properly. And most zygotic splicing factors were not activated in the 2‐cell stage (Figure 2E,F). The RT‐qPCR results verified this conclusion (Figure S5A,B, Supporting Information), indicating that the regulation of splicing factor expression during MZT is abnormal in *Btg4^−/−^* and *Pabpn1l ^−/−^* oocytes.

In addition to the impairment of M‐decay caused by the deletion of parental factors during MZT, the inability of ZGA to be properly activated is also a distinctive feature. α‐Amanitin is a transcription inhibitor that selectively inhibits RNA polymerase II and III.^[^ ^46^ ^]^ After treating the zygote with α‐Amanitin, we observed a decrease in SRSF2 expression at the 2‐cell stage, accompanied by the disappearance of its speckle‐like aggregation (Figure S5C, Supporting Information). α‐Amanitin‐treated zygotes are unable to activate zygotic splicing (Figure S5D,E, Supporting Information). The activation of alternative splicing fails when transcription is repressed, further suggesting that transcriptional activation is the primary driver of alternative splicing. Based on these results, we found that the aberrant expression of splicing factors due to maternal factor deletion and the impairment of ZGA during MZT can cause the failure of alternative splicing activation.

### 2.3 PlaB as a Splicing Factor Disruptor Can Affect Early Embryonic Development

**Figure 3:**
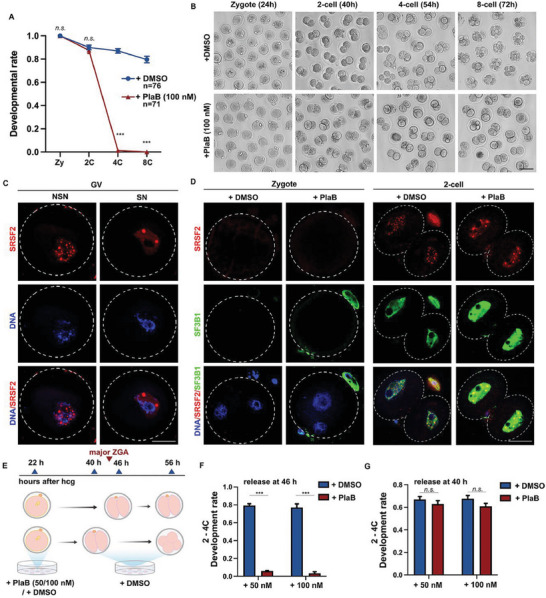
Zygotic splicing activation failure leads to the arrest of embryonic development at 2‐cell stage. A) The development rates of preimplantation embryos after treatment with pladienolide B (PlaB; 100 nm) or dimethylsulfoxide (DMSO) when embryos reached corresponding stages. The numbers of analyzed embryos are indicated (n). *n* = 3 biological replicates. Error bars, standard error of the mean (SEM); n.s.: non‐significant. ^***^ *p* < 0.001 by two‐tailed Student's *t*‐test. B) Representative images of preimplantation embryos at different stages after treatment with PlaB (100 nm). Scale bar, 100 µm. Time after human chorionic gonadotropin (hCG) injection is indicated (h). C) Immunofluorescent staining showing the speckles of SRSF2 and DAPI in oocytes at non‐surrounded nucleolus (NSN) and surrounded nucleolus (SN) stages. Scale bar, 20 µm. D) Immunofluorescent staining showing the speckles of SRSF2 and the expression level of SF3B1 in zygote and 2‐cell embryos after treatment with PlaB (100 nm) or DMSO. Scale bar, 20 µm. E) Illustration of the time points when the samples were released for experiments in (F) and (G). F,G) The 2–4‐cell development rates of preimplantation embryos after release from DMSO or PlaB treatment at different time points. Error bars, SEM; n.s.: non‐significant. ^***^ *p* < 0.001 by two‐tailed Student's *t*‐test.

Dynamic changes in alternative splicing during MZT in mouse embryos suggest that the activation of zygotic splicing plays an important role in early embryonic development. PlaB, a competitive inhibitor of the key splicing factor SF3B1,^[^ ^27^ ^]^ was added to the culture media of mouse oocytes and early embryos (Figure S6B,C, Supporting Information). PlaB had no significant effect on meiotic resumption in oocytes (Figure S6D,E, Supporting Information), but concentrations above 50 nm caused significant arrest in embryonic development at the 2‐cell stage (Figure S6F, Supporting Information). When the control embryos developed to the 8‐cell stage, PlaB‐treated embryos remained arrested at the 2‐cell stage, and very few embryos developed to the 4‐cell stage (**Figure** **3**A,B).

SRSF2, a widely studied splicing factor, has previously been reported to exhibit punctate aggregation in growing oocytes, disappear during the zygote stage, and reappear during the 2‐cell stage.^[^ ^33^ ^]^ This process of speckle‐like aggregate rebuilding in early embryos also indicates the reactivation of alternative splicing. Therefore, we performed immunofluorescent (IF) staining of SRSF2 in GV oocytes and classified them into non‐surrounded nucleolus (NSN) or surrounded nucleolus (SN) oocytes according to their chromatin conformation.^[^ ^47^ ^]^ At the GV stage, SN oocytes are characterized by transcriptional silencing when splicing activity is minimal, whereas transcription and splicing are still active in NSN oocytes. We found that SRSF2 still showed many small speckles in NSN oocytes, but the speckles became larger and fewer in SN oocytes, which reflected the localization state of SRSF2 when alternative splicing was inactive (Figure 3C). During the ZGA process, the transcript levels of SRSF2 and SF3B1 are not high, but their translation levels significantly increase (Figure S7A,B, Supporting Information). SF3B1 protein is expressed at the 2‐cell stage, and SRSF2 exhibits distinct speckle‐like aggregation from the 2‐cell to the 8‐cell stages. This indicates that following the initiation of ZSA, alternative splicing remains actively engaged throughout early embryonic development (Figure S7C,D, Supporting Information). SRSF2 showed punctate localization, but SF3B1 was evenly distributed in the nuclei (Figure 3D). After PlaB treatment, SRSF2 speckles decreased in number but increased in size (Figure 3D; Figure S7E, Supporting Information), whereas the nuclear expression signals of SF3B1 became more significant, possibly due to a feedback regulation mechanism after the competitive binding of SF3B1 by PlaB (Figure 3D).

To determine the precise timing and mechanism of action of PlaB, we conducted a release experiment (Figure 3E). When released at 40 h after human chorionic gonadotropin (hCG) administration, the PlaB group successfully restored the 2‐ to 4‐cell development rate to that of the control group. However, the release of embryos at 46 h did not rescue the arrested phenotype (Figure 3F,G). These findings indicate that PlaB exerts its effects during the major ZGA stage, specifically between 40 and 46 h after hCG administration. PlaB interferes with the ZSA process, thereby affecting embryonic development.

### 2.4 Activation of SF3B1 Phosphorylation Is Necessary for Efficient Splicing

**Figure 4:**
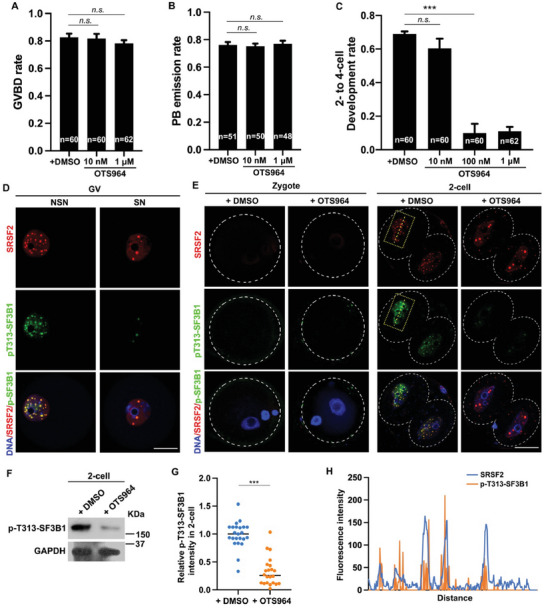
Phosphorylation of SF3B1 at the 2‐cell stage is essential for zygotic splicing activation. A) Comparison of germinal vesicle breakdown (GVBD) rates in cultured oocytes treated with dimethyl sulfoxide (DMSO) or OTS964. B) The rates of polar body emission (PBE) in cultured oocytes treated with DMSO or OTS964. When oocytes had undergone GVBD within 6 h, they were selected for further culture. C) The development rates of 2–4‐cell embryos after treatment with DMSO or OTS964. The numbers of analyzed embryos are indicated (n). *n* = 3 biological replicates. Error bars, SEM; n.s.: non‐significant. ^***^ *p* < 0.001 by two‐tailed Student's *t*‐test. D) Immunofluorescent staining showing the speckles of SRSF2 and the levels of p‐T313‐SF3B1 in oocytes at the NSN and SN stages. Scale bar, 20 µm. E) Immunofluorescent staining showing the speckles of SRSF2 and the expression levels of SF3B1 in zygotes and 2‐cell embryos after treatment with PlaB (100 nm) or DMSO. Scale bar, 20 µm. F) Western blotting results of p‐T313‐SF3B1 in 2‐cell embryos after treatment from the zygote stage with DMSO or OTS964. G) Quantification of p‐T313‐SF3B1 signal intensity in (C). ^***^ *p* < 0.001 by two‐tailed Student's *t*‐test. H) ImageJ was used to quantitatively analyze the distribution of SRSF2 and p‐T313‐SF3B1 in (C), indicated by a yellow dotted line within the square frame.

OTS964, a small‐molecule inhibitor, suppresses SF3B1 phosphorylation by targeting CDK11 kinase activity.^[^ ^29^ ^]^ Although there was no significant effect on oocyte maturation (**Figure** **4**A,B), concentrations above 100 nm led to developmental blockade at the 2‐cell stage, which was consistent with the PlaB‐treatment phenotype (Figure 4C). Consistent with the expression patterns of SRSF2 and SF3B1, CDK11 and its associated cyclin, Cyclin L1, also exhibit low RNA expression levels during the ZGA process, but show an increase in translation levels (Figure S7F,G, Supporting Information). IF staining of p‐T313‐SF3B1 was performed in GV oocytes and early embryos at different time points, demonstrating the reconstitution of SF3B1 phosphorylation during alternative splicing activation (Figure 4D,E). Western blotting and IF results confirmed the effective inhibition of SF3B1 phosphorylation by OTS964 in mouse embryos (Figure 4E,F). Treatment with OTS964 inhibited SF3B1 phosphorylation and altered SRSF2 localization, indicating disrupted alternative splicing (Figure 4G; Figure S7H, Supporting Information). Furthermore, IF revealed strong colocalization of p‐T313‐SF3B1 with the SRSF2 signal, supporting the requirement of SF3B1 activation through phosphorylation for its functional role, as evidenced by the upregulation of SF3B1 after PlaB treatment (Figure 4E,H).

These results indicate that PlaB and OTS964 disrupt splicing by targeting the splicing factor SF3B1. The phosphorylation of SF3B1 by CDK11 is essential for the activation of splicing, and its co‐localization with SRSF2 in patchy aggregates occurs after phosphorylation.

### 2.5 PlaB Disrupts Alternative Splicing, but Does Not Affect Transcriptional Activity

**Figure 5:**
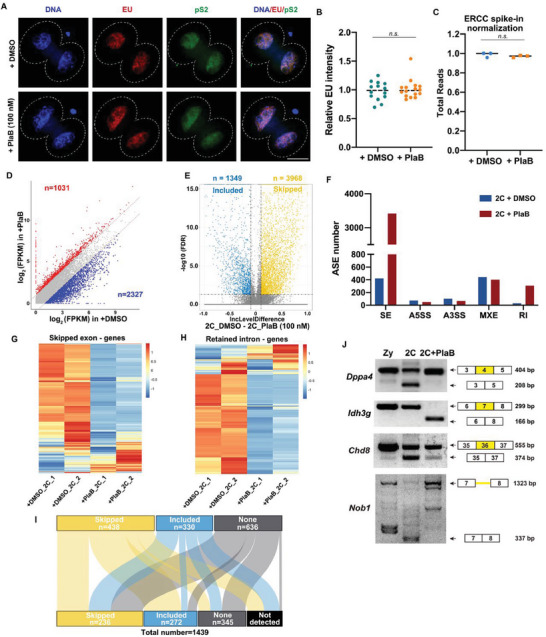
Dynamic changes in transcript isoforms and expression levels in embryos treated with PlaB. A) Immunofluorescent staining of 5‐ethynyl uridine (EU) and pS2 fluorescence showing RNA transcription in 2‐cell embryos treated with PlaB or DMSO. B) Quantification of EU signal intensity in (A). Error bars, SEM; n.s.: non‐significant. C) Total mRNA quantification of 2‐cell embryos treated with PlaB or DMSO. The results were normalized to an External RNA Controls Consortium (ERCC) spike‐in. Error bars, SEM; n.s.: non‐significant. D) Scatter plot comparing the expressing level of transcripts between 2‐cell embryos treated with PlaB or DMSO. Transcript levels that decreased or increased by more than twofold in 2‐cell embryos treated with PlaB are highlighted in blue or red, respectively. E) Volcano plot showing changes in transcript isoforms skipped or included in 2‐cell embryos treated with PlaB or DMSO. F) Change in the numbers of five different ASEs in 2‐cell embryos treated with PlaB or DMSO. G,H) Heatmap of genes showing the normalized expression levels of transcripts with a skipped exon (SE) or an intron retained (IR) in 2‐cell embryos treated with PlaB. The value in the figure represents the relative mRNA level. I) The global changes of genes during the zygote to 2‐cell undergoing differential SE events after treatment with PlaB. Top column: genes undergoing differential SE events during the zygote to 2‐cell. Lower column: genes undergoing differential SE events during 2‐cell stage after treatment with PlaB. Each bar represents the class of differential SE events. Skipped: More exons skipped (ILD>0.1, FDR<0.05); Included: More exons included (ILD←0.1, FDR<0.05); none: showing no difference; not detected: genes not detected during the 2‐cell stage after treatment with PlaB. J) Reverse transcription‐polymerase chain reaction analysis results of different transcript isomers of genes after PlaB treatment.

Our results have found that ZSA relies on the MZT process. However, the effects of using PlaB to disrupt splicing in the transcriptome and ZSA remain unclear. We detected global transcription activities in the 2‐cell embryos using a 5‐ethynyl uridine (EU) incorporation assay and IF staining of RNA polymerase II phosphorylated at serine‐2 (pS2) and found that the transcriptional activity was minimally affected (**Figure** **5**A,B). Additionally, the overall quantity of the transcripts did not change significantly upon treatment with PlaB (Figure 5C). However, when examining specific transcripts using scatter plots, we observed a greater number of transcripts with lower expression levels (Figure 5D). Considering the absence of notable changes in transcriptional activity, this subset of transcripts may be attributed to the degradation pathway. To validate this hypothesis, we analyzed alternative splicing alterations after PlaB treatment and discovered a substantial increase in new ASEs (Figure 5E). Categorical analysis of these events revealed a predominant increase in two categories, SE and IR, with an approximately seven‐fold increase for each (Figure 5F). Further examination of the expression levels within this subset of transcripts exhibiting SE and IR revealed predominantly downregulated transcript levels (Figure 5G,H).

After PlaB administration, we observed a significant increase in the number of ASEs. To investigate the impact of PlaB on alternative splicing, we traced genes undergoing alternative splicing from the zygote to the 2‐cell stage using a river plot. We found that regardless of the splicing events – whether exon skipping, exon retention, or genes without significant splicing events – there was a dramatic change in the type of splicing after PlaB treatment (Figure 5I). This suggests that PlaB treatment disrupts the recognition of intron branch points, leading to interference with alternative splicing. Several ASEs were selected for validation. *Dppa4*, a gene associated with early embryonic development, displays partial skipping of exon 3 and exon 4 at the 2‐cell stage. Similarly, *Chd8*, which encodes a DNA‐binding protein, skips exon 36 at the 2‐cell stage. These SE events were suppressed by PlaB treatment. In contrast, *Idh3g*, which encodes an isoconjugate dehydrogenase, did not undergo alternative splicing during the 2‐cell stage, but skipped exon 7 after PlaB treatment. Another gene, *Nob1*, which is involved in ribosome assembly, demonstrated splicing of the intron between exon 7 and exon 8 at the 2‐cell stage, whereas IR occurred after PlaB treatment (Figure 5J).

Our findings indicated that PlaB treatment disrupts transcriptome homeostasis in early embryos. This disruption is primarily attributed to the perturbation of alternative splicing, resulting in an increase in the number of ASEs and a decrease in the number of suppressed ASEs.

### 2.6 RNA Isoforms Resulting from Alternative Splicing Are Necessary for Embryonic Development

**Figure 6:**
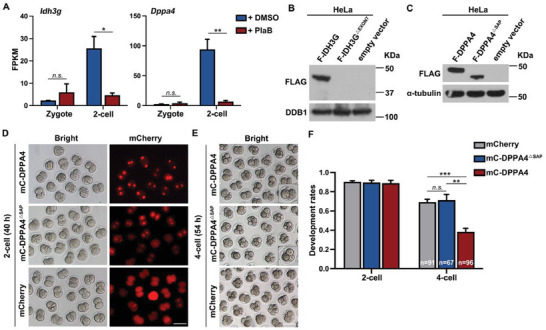
Impact of *Dppa4* alternative splicing on preimplantation mouse embryo development. A) FPKM values of *Idh3g* and *Dppa4* derived from RNA‐seq data. B) The western blotting results confirmed that the deletion of exon 7 of *Idh3g* resulted in abnormal protein expression. C) The western blotting results confirmed that the deletion of the SAP domain of *Dppa4* resulted in the translation of a smaller protein isoform. D) Fluorescence microscopy results showing mCherry‐DPPA4 (mC‐DPPA4) and mC‐DPPA4^△SAP^ expression in 2‐cell stage embryos (40 h after hCG). E) Representative images of preimplantation embryos at 4‐cell stage (54 h after hCG). Scale bar, 100 µm. F) Development rates of cultured control and mC‐DPPA4^△SAP^‐ and mC‐DPPA4‐overexpressing zygotes. Error bars, SEM; n.s.: non‐significant. ^*^ *p* < 0.05, ^**^ *p* < 0.01, ^***^ *p* < 0.001 by two‐tailed Student's *t*‐test.

Alternative splicing of mRNA can lead to two outcomes: the production of distinct isoforms with different functions or the generation of premature stop codons that trigger degradation via the NMD pathway. Multiple isoforms are often present simultaneously during embryonic development. The isoform that functions correctly during this stage and promotes embryonic development is classified as the plus (+) isoform. Conversely, the isoform that hinders embryonic development and requires degradation is referred to as the minus (‐) isoform. Further investigation was conducted to examine how these distinct isoforms affect transcript stability and embryonic development. In both *Idh3g* and *Dppa4*, exon skipping significantly reduced transcript levels (**Figure** **6**A). Specifically, an exon 7 jump in *Idh3g* resulted in a frameshift mutation, rendering proper translation of the protein impossible (Figure 6B). The SAP domain, which is a DNA‐binding motif, spans exons 3 and 4 of *Dppa4*. Deletion of this structural domain resulted in a shorter form of the protein isoform (Figure 6C).

To assess the effect of alternative splicing on early embryonic development, we injected two distinct isoforms of *Dppa4* along with empty mCherry plasmids into zygotes after performing in vitro mRNA transcription. Embryos injected with full‐length *Dppa4* exhibited normal development until the 2‐cell stage. However, the rate of development from 2‐cell stage to 4‐cell stage was significantly lower than the rate of development of embryos injected with *Dppa4* lacking the SAP domain (Figure 6F).

In general, different isoforms generated by alternative splicing have distinct outcomes and effects. Isoforms that undergo frameshift mutations, such as *Idh3g*, cannot be translated properly, and are degraded via the NMD pathway. In contrast, plus isoforms that do not experience frameshift mutations are beneficial or have no significant impact on early embryonic development. Conversely, the minus isoforms hinder embryonic development, similar to *Dppa4*. These findings further illustrate the critical importance of proper ZSA during early embryonic development, because isoforms produced by erroneous splicing impede development.

### 2.7 Alternative Splicing Affects the Totipotent to Pluripotent Transition of Early Embryos

**Figure 7:**
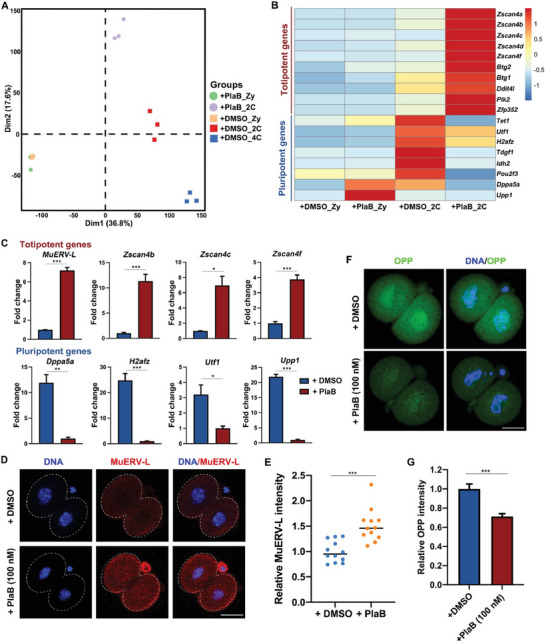
Effect of PlaB treatment on totipotent and pluripotent gene expression levels in 2‐cell embryos. A) Transcriptome‐based PCA of embryos treated with PlaB or DMSO. B) Heatmap of the relative expression levels of representative totipotent and pluripotent genes in 2‐cell embryos treated with PlaB or DMSO. C) RT‐qPCR validation of the expression levels of totipotent and pluripotent genes in 2‐cell embryos treated with PlaB or DMSO. Error bars, SEM. ^*^ *p* < 0.05, ^**^ *p* < 0.01, ^***^ *p* < 0.001 by two‐tailed Student's *t*‐test. D) Immunofluorescent staining showing the level of MuERV‐L in 2‐cell embryos treated with PlaB or DMSO. Scale bar, 20 µm. E) Quantification of MuERV‐L signal intensity in (F). ^***^ *p* < 0.001. F) O‐propargyl‐puromycin (OPP) staining results showing protein synthesis activity at zygote and 2‐cell stages in 2‐cell embryos treated with PlaB or DMSO. Before staining, these embryos were incubated in a medium containing 20 µm OPP for 30 min. Scale bar, 20 µm. G) Quantification of OPP signal intensity in (D). Error bars, SEM. ^***^ *p* < 0.001.

In a previous study, PlaB was shown to transform 2‐cell‐like cells into pluripotent cells, referred to as TBLCs.^[^ ^28^ ^]^ Our findings indicated that PlaB induces developmental blockage at the crucial 2‐cell stage in early embryos when the transition from totipotency to pluripotency occurs. To investigate this relationship, we initially observed distinct differences between PlaB‐treated and control 2‐cell embryos using principal components analysis (PCA) (**Figure** **7**A). Transcriptomic analysis revealed a notable upregulation of specific totipotent genes expressed in zygotes and 2‐cell embryos, including *Zscan4s*, *Btg1/2*, *Ddit4l*, *Zfp352*, and the transposon *MuERV‐L*, whereas the expression of typical pluripotent genes, such as *Dppa5a*, *H2afz*, *Utf1*, and *Upp1*, was significantly downregulated in PlaB‐treated 2‐cell embryos (Figure 7B). This finding was further validated by RT‐PCR (Figure 7C) and IF staining of MuERV‐L, which showed abnormally high expression levels of totipotent genes (Figure 7D,E). Additionally, we assessed the impact of altered transcriptional expression on translational activity using an O‐propargyl‐puromycin (OPP) kit and observed a significant decrease in the translational activity of PlaB‐treated 2‐cell embryos (Figure 7F,G). Collectively, these results suggest that ZSA plays a role in the conversion of totipotency to pluripotency in early embryos.

## 3 Discussion

MZT is a critical event during early embryonic development. It involves the degradation of maternal mRNA, which accumulates in large quantities, and the subsequent activation of the zygotic genome, resulting in mRNA renewal. Despite the significance of MZT, there are numerous unknown regulatory mechanisms governing mRNA expression during this process. Investigating these mechanisms can enhance our understanding of the vital roles of mRNAs in early embryonic development and contribute to resolving reproductive challenges associated with embryonic development.

Our analysis of previously published RNA‐seq data from early mouse and human embryos revealed a consistent and dynamic trend in alternative splicing during mammalian embryonic development. We observed significant activation of alternative splicing, referred to as ZSA, in the ZGA stage. Through cluster analysis, we observed stage‐specificity in different exon‐skipping events during early embryonic development. We further categorized these into two distinct groups with pronounced splicing characteristics: peak‐like and shift‐like clusters. Peak‐like AS events are transient and specific to the window of ZGA, whereas shift‐like AS events reflect the fate transition from maternal control to zygotic control. Further analysis of RNA‐seq data from embryos with maternal factors (*Btg4* and *Pabpn1l*) knocked out and those treated with the transcriptional repressor amanitin demonstrated that appropriate expression of splicing factors and normal transcriptional activation is crucial for splicing activation. Based on our findings, we propose a hypothesis that during MZT, mRNAs undergo not only changes in gene expression patterns but also transformations in isoform diversity through alternative splicing. The accurate expression of an appropriate isoform is essential for proper embryonic development. Additionally, when embryos were exposed to splicing‐disrupting drugs, such as PlaB and OTS964, mouse embryo development was arrested at the 2‐cell stage, indicating the significance of the splicing process in embryo development.

We observed dynamic changes in the splicing factors SRSF2 and SF3B1, both of which are crucial to the alternative splicing process.^[^ ^48^, ^49^ ^]^ We identified the process of splicing factor reactivation during the development of zygotes into 2‐cell embryos, demonstrating the activation of alternative splicing at the ZGA stage. Previous studies have also demonstrated that the small‐molecule drugs PlaB and OTS964 can competitively bind SF3B1 or inhibit SF3B1 phosphorylation in somatic cells, respectively, thereby influencing alternative splicing.^[^ ^25^, ^26^, ^27^ ^]^ We explored the roles of splicing factors in early embryos. The inhibition of SF3B1 mRNA binding or phosphorylation disrupts normal embryonic development and is closely associated with ZGA. Alternative splicing relies on transcriptional activation, but does not directly impact transcription. This suggests that SF3B1‐mediated alternative splicing serves as a post‐transcriptional regulatory modification that influences the expression and function of transcripts through code‐shift mutations and partial silencing. This disruption has a significant impact on early embryonic development. Additionally, our study demonstrated that alternative splicing in early mouse embryos can influence the transition from totipotency to pluripotency and affect the overall translational activity of the embryo. However, the specific regulatory mechanisms underlying this process require further investigation.

From the above results, we obtained some interesting conclusions, which are discussed below. Transcriptional activation at specific developmental time points is a common feature of early mammalian embryonic development, and splicing is necessary for precursor mRNA processing accompanied by transcriptional activation. Additionally, alternative splicing, a specific mode of splicing that regulates gene expression and protein isoform function, undergoes dynamic changes during the ZGA stage, suggesting its vital role in early embryonic development. Similarly, our results showed that interference with splicing factors using inhibitors directly affects alternative splicing, but not transcriptional activity, and there was no significant change in total mRNA in 2‐cell embryos after dimethylsulfoxide (DMSO) or PlaB treatment. This indicates that, during early embryonic development, transcriptional activation of the zygotic genome provides a substantial amount of material for alternative splicing. Alternative splicing plays a crucial role in regulating transcript stability and generating isoforms, collectively ensuring the normal development of early embryos.

The ZSA stage generates numerous transcriptional isoforms. Our hypothesis suggests that alternative splicing functions as a regulatory mechanism for partial silencing. This mechanism enables the selective retention or skipping of functional domains in mRNAs, thereby maintaining certain functions while silencing others. A recent study suggested that during the ZGA stage of early human embryonic development, alternative splicing activity is vigorous and disrupts open reading frames in some transcripts, resulting in their expression termination. This situation reversed at the blastocyst stage, hence leading to developmental stage‐specific gene expressions regulated by alternative splicing.^[^ ^50^ ^]^ The results we presented in this study are consistent with these findings, suggesting that some genes are specifically silenced during the ZGA process in mice embryos by an alternative splicing‐dependent mechanism. Therefore, it may play a significant role in embryonic development. Our experimental results revealed that the full‐length isoform of *Dppa4* leads to developmental arrest at the 2‐cell stage gene. Additionally, previous studies of *Dppa4*‐knockout mice have indicated that deleting the *Dppa4* gene does not significantly affect development at the 2–4 cell stage.^[^ ^51^, ^52^ ^]^

In our previous research, we found that knocking out maternal factors, such as *Btg4* and *Pabpn1l*, leads to the developmental arrest of mouse early embryos at the zygote to 2‐cell stage. However, the molecular mechanisms underlying this phenomenon are not fully understood. Our analysis of alternative splicing during MZT revealed that ZSA failure may be one of the reasons for developmental impairment in mouse embryos lacking BTG4 and PABPN1L. Knocking out the maternal factors *Btg4* and *Pabpn1l*, which are associated with M‐decay, disrupts numerous splicing factors. This leads to the improper degradation of maternal splicing factors and inadequate activation of zygotic splicing factors. These findings indicate that splicing factors undergo a transition from the maternal to the zygotic form during MZT. We found that the disruption of splicing factor expression due to M‐decay or the failure of ZGA during MZT resulted in the failure of ZSA. This further suggests that the MZT process prepares mice for the normal activation of alternative splicing during early embryonic development.

Embryonic development was arrested at the 2‐cell stage after interfering with SF3B1 using the small‐molecule inhibitor PlaB, further indicating that SF3B1‐mediated alternative splicing plays an important role in early embryonic development. Notably, our observations on SRSF2 indicated different splicing domain states in GV oocytes with varying chromatin configurations, reflecting the active or silent nature of splicing. During ZSA, we noticed the re‐establishment of an active splicing domain, which was surprising given that SF3B1 did not co‐localize with SRSF2, but instead, showed a diffuse distribution within the nucleus. We investigated the localization of phosphorylated SF3B1 and discovered strong co‐localization with SRSF2 at different stages, suggesting their collaborative role in the splicing domain. Additionally, we found that the inhibitor, OTS964, which targets CDK11, significantly impedes early embryonic development and leads to a shift in the splicing domain to a silent state by inhibiting SF3B1 phosphorylation. These results imply that the CDK11‐mediated activation of SF3B1 phosphorylation is necessary for ZSA.

Recent studies have revealed that alternative splicing regulates spectral differentiation and the state transition of embryonic stem cells.^[^ ^53^, ^54^ ^]^ Using PlaB to culture embryonic stem cells, it was found that interference with alternative splicing converts embryonic stem cells to a totipotent state, resulting in the culture of more totipotent embryonic stem cells known as TBLCs. This study posits that the reason totipotency genes can still be expressed despite splicing errors is due to their having fewer and shorter introns.^[^ ^28^ ^]^ During early mouse embryonic development, the 2‐cell stage is considered a pivotal time point for the transition from totipotency to pluripotency. Therefore, we examined the effect of PlaB on the switch from totipotency to pluripotency in early mouse embryos. Consistent with our previous findings, we observed abnormal activation of totipotent genes and the suppression of pluripotent gene expression, including significantly heightened activation of the retrotransposon MUERVL. We observed similar effects of PlaB in different cell types and predicted that alternative splicing plays an important regulatory role in the switch between totipotency and pluripotency, thereby affecting embryonic lineage differentiation. However, current research still does not fully explain the mechanism by which alternative splicing affects the transition between totipotency and pluripotency in early embryos. This remains an interesting and important direction for future studies.

**Figure 8:**
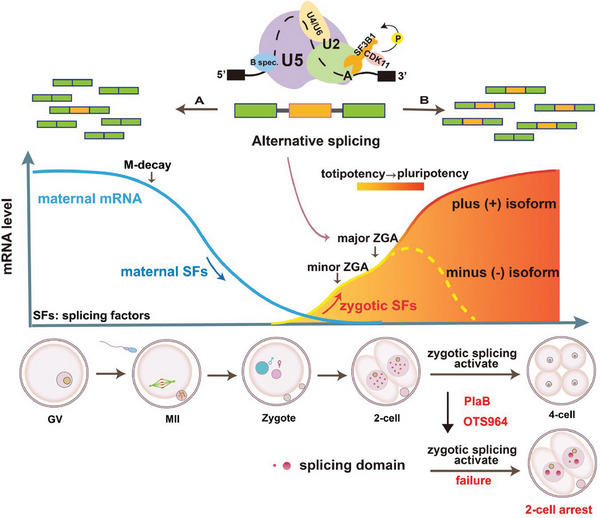
Summary of the pattern and functions of alternative splicing during the development of preimplantation mouse embryos and zygotic genome activation. mRNA undergoes complex regulation during oocyte maturation and early embryonic development, including M‐decay and zygotic genome activation (ZGA). Maternal mRNA (including maternal splicing factors) follows a degradation trend represented by the blue curve. The red‐yellow curve represents the activation of zygotic transcripts (including zygotic splicing factors), with the transition from yellow to red indicating the conversion of early embryos from totipotency to pluripotency. During this process, alternative splicing is activated to produce different isoforms, ensuring normal embryonic development by allowing the function of the plus isoform, while degrading the minus isoform. Zygotic splicing activation (ZSA) involves the formation of multiple splicing domains by the splicing factor SRSF2, while treatment with SF3B1 inhibitors results in fewer and larger splicing domains, leading to arrested embryonic development at the 2‐cell stage.

In summary, our study revealed the dynamic changes in alternative splicing during early embryonic development. We proposed the concept of ZSA for the first time and discovered the transition process of splicing factors from maternal to zygotic control. Maternal mRNA degradation and zygotic activation provide the driving force for these processes, while alternative splicing further processes the mRNA, generating appropriate isoforms that facilitate the transition of early embryos from pluripotency to multipotency and ensure smooth development (**Figure** **8**). Additionally, we highlighted the physiological significance of alternative splicing in early embryonic development in mice and examined the mechanism underlying embryonic developmental arrest resulting from disruptions in alternative splicing.

## 4 Experimental Section

### Mice

Wild‐type (WT) ICR mice were purchased from the Zhejiang Academy of Medical Science, China. The mice were housed under specific‐pathogen‐free conditions in a constant environment, including a temperature of 20–22 °C, a 12/12‐h light/dark cycle, 50–70% humidity, and food and water provided ad libitum. Animal care and experimental procedures were conducted in accordance with the guidelines of the Animal Research Committee of Zhejiang University.

### Oocyte Culture

Three‐week‐old female mice were intraperitoneally primed with 5 IU of pregnant mare serum gonadotropin (PMSG) and were humanely euthanized after 44 h. NSN and SN oocytes were all collected from antral follicles and at the fully grown stage. Fully grown oocytes were harvested in M2 medium (M7167; Sigma–Aldrich) and cultured in mini‐drops of M16 medium (M7292; Sigma–Aldrich) covered with mineral oil (M5310; Sigma–Aldrich) and then incubated at 37 °C in a 5% CO_2_ atmosphere.

### Superovulation and Fertilization

Three‐week‐old female mice were intraperitoneally injected with 5 IU PMSG. After 44 h, the mice were injected with 5 IU of hCG. After an additional 16 h, oocyte–cumulus complexes were retrieved from the oviducts, and oocytes were obtained after treatment with hyaluronidase (Sigma–Aldrich). To obtain early embryos, female mice were mated with 10–12‐week‐old wild‐type (WT) males. Successful mating was confirmed by the presence of vaginal plugs. Embryos were harvested from oviducts at the indicated time points after hCG injection.

### Treatment of Mouse Embryos with PlaB or OTS964

Zygotes were collected from oviducts 22 h after hCG treatment. To disturb splicing in early embryos, zygotes were cultured in potassium simplex optimized medium (KSOM) supplemented with PlaB (Chemegen, Shanghai, China; Cat. No.: CY14134) or OTS964 (Med Chem Express, Monmouth Junction, NJ, USA; Cat. No.: HY‐19718). After culturing, morphologically normal 2‐cell embryos were collected for additional experiments.

### Plasmid Construction and In Vitro Transcription

The corresponding cDNA was subcloned into an mCherry‐tagged expression vector (pDEST). The expression vector was linearized with HindIII, and the linearized RNA was transcribed in vitro using a T7 mMESSAGE mMACHINE kit (Invitrogen, Carlsbad, CA, USA; AM1344). Phenol/chloroform and ethanol precipitation were used for RNA extraction and purification, respectively. Transcribed RNAs were in vitro polyadenylated using a poly (A) tailing kit (AM1350; Invitrogen).

### Microinjection of mRNAs

All microinjections were performed using an Eppendorf TransferMan NK2 micromanipulator. Fully grown oocytes were cultured in M2 medium containing 2 µm milrinone to suppress spontaneous germinal vesicle breakdown. Approximately 5–10 pL of 500 g mL^−1^ mRNA was microinjected into the oocyte cytoplasm. As a negative control, 5–10 pL of 500 µg mL^−1^ RNA transcribed from the empty vector (pDEST‐mCherry) was microinjected into the control oocytes. The oocytes were cultured in M16 medium containing 2 µm milrinone at 37 °C in a 5% CO_2_ atmosphere.

### EU Incorporation Assay

Embryos were cultured in KSOM containing 100 µm EU for 2 h. Fixation, permeabilization, and staining were performed using the Click‐iT RNA Alexa Fluor 488 Imaging Kit (Thermo Fisher Scientific, Waltham, MA, USA; 48 C10329) according to the manufacturer's protocol. The embryos were imaged using a Zeiss LSM710 confocal microscope (Zeiss, Oberkochen, Germany).

### Detection of Protein Synthesis

Embryos at the 2‐cell stage were incubated in KSOM supplemented with 20 µm Click‐iT OPP (O‐propargyl‐puromycin) for 30 min. The embryos were then fixed for 30 min in 4% paraformaldehyde (PFA). OPP signals were examined using the Click‐iT Plus OPP Protein Synthesis Assay Kit (Life Technologies, Carlsbad, CA, USA). The mean OPP signal was measured and quantified using ImageJ software (National Institutes of Health, Bethesda, MD, USA).

### Western Blotting Analysis

Oocytes were lysed in a loading buffer containing β‐mercaptoethanol and then heated at 95 °C for 10 min. Immunoblotting and sodium dodecyl sulfate–polyacrylamide gel electrophoresis were performed following standard procedures using the Mini‐PROTEAN Tetra Cell System (Bio‐Rad, Hercules, CA, USA). All antibodies and their dilution factors are listed in Table S1 (Supporting Information).

### Quantitative RT‐PCR

Total RNA was extracted from oocytes using the RNeasy Mini Kit (QIAGEN, Hilden, Germany) according to the manufacturer's instructions. Reverse transcription was performed using a Superscript RT kit (Bio‐Rad). Quantitative RT‐PCR was conducted using Power SYBR Green PCR Master Mix (Applied Biosystems, Life Technologies) on an ABI 7500 Real‐Time PCR System (Applied Biosystems, Waltham, MA, USA). The primers used are listed in Table S2 (Supporting Information).

### Immunofluorescence

Oocytes and embryos were fixed with 4% PFA for 30 min and permeabilized for 20 min with 0.3% Triton X‐100 in phosphate‐buffered saline. Antibody staining was performed according to previously described protocols.^[^ ^55^ ^]^ The antibodies used in these experiments are listed in Table S1 (Supporting Information). Imaging was performed using a Zeiss LSM710 confocal microscope. Quantitative analysis of the fluorescence signals was performed using ImageJ software.

### RNA‐Seq Library Preparation and Data Analysis

Oocytes or embryos were collected from 3‐week‐old WT female mice (10 oocytes or embryos per sample). External RNA Controls Consortium (ERCC) molecules (3 × 10^7^) were added to the lysis buffer. Total RNA was extracted using the RNeasy micro kit (QIAGEN) following the manufacturer's instructions after picking the cells into 350 mL of the lysis buffer supplied in the kit. Libraries were prepared using an NEBNext Ultra RNA Library Prep Kit (Illumina, San Diego, CA, USA). The libraries were sequenced on an Illumina platform with 150‐bp paired‐end reads. A script was used to filter out low‐quality reads, and clean reads were mapped to the mouse genome 10 mm using TopHat (version 2.0.6). Gene expression levels were calculated and normalized to FPKM using cuffquant and cuffnorm. The absolute mRNA copy number was calculated using the spiked‐in ERCC molecules. FPKM and mRNA copy number values were used to represent gene expression levels. Only the expressed genes (FPKM > 1 in at least one sample) were considered in subsequent analyses.

### Analysis of Various Alternative Splicing Events

Using replicate multivariate analysis of transcript splicing (rMATS),^[^ ^56^, ^57^ ^]^ five different ASEs: SE, A5SS, A3SS, mutually exclusive exons (MXE), and intron retention were detected. Various alternative splicing events were considered when the false‐discovery rate (FDR) was < 0.05 and the IncLevelDifference was > 0.1 or < −0.1.

### Splicing Factor Analysis

The list of total splicing factors was derived from published articles and “RNA splicing”‐ or “spliceosome”‐associated GO terms from MGI.^[^ ^35^, ^43^, ^44^, ^45^ ^]^ All splicing factors, as well as maternal and zygotic splicing factors associated with *Btg4*‐ and *Pabpn1l*‐knockout mice, are listed in Table S3 (Supporting Information).

### Data Availability

The datasets generated during the current study are available in the National Center for Biotechnology Information repository with the accession number PRJNA004899, https://dataview.ncbi.nlm.nih.gov/object/PRJNA1004899?reviewer = 4c6uu475q5le2gpbjcu5i35tf0. Previously published RNA‐seq data^[^ ^6^, ^42^, ^58^, ^59^, ^60^ ^]^ used in this work were from NCBI GEO accession number GSE174032 and GSE179406 (mouse oocytes and embryos), GSE44183 (human oocytes and embryos), GSE71434 (α‐amanitin treated embryos), GSE165782(Smart‐seq2 and Ribo‐seq).

## Conflict of Interest

The authors declare no conflict of interest.

## Supporting information

Supporting Information

Supplemental Table 1

Supplemental Table 2

Supplemental Table 5

## Data Availability

The data that support the findings of this study are available from the corresponding author upon reasonable request.
