# Supplementary material for: Zygotic Splicing Activation of the Transcriptome is a Crucial Aspect of Maternal‐to‐Zygotic Transition and Required for the Conversion from Totipotency to Pluripotency
Source: Adv Sci (Weinh). 2024 Feb 2;11(14):2308496. doi: 10.1002/advs.202308496 (PMC11005748; doi:10.1002/advs.202308496)
Supplement: Supplementary file 1 — Supporting Information [file ADVS-11-2308496-s001.pdf]

ADVANCED SCIENCE Open Access

Supporting Information

for *Adv. Sci.*, DOI 10.1002/advs.202308496

Zygotic Splicing Activation of the Transcriptome is a Crucial Aspect of Maternal-to-Zygotic Transition and Required for the Conversion from Totipotency to Pluripotency

Hua Zhang, Yang Wang, Zhe-Wei Hu, Yun-Wen Wu, Nuo Chen, Yi-Min Zhu, Yuan-Song Yu, Heng-Yu Fan and Hua-Nan Wang**

Supplementary Information

Supplementary Figures

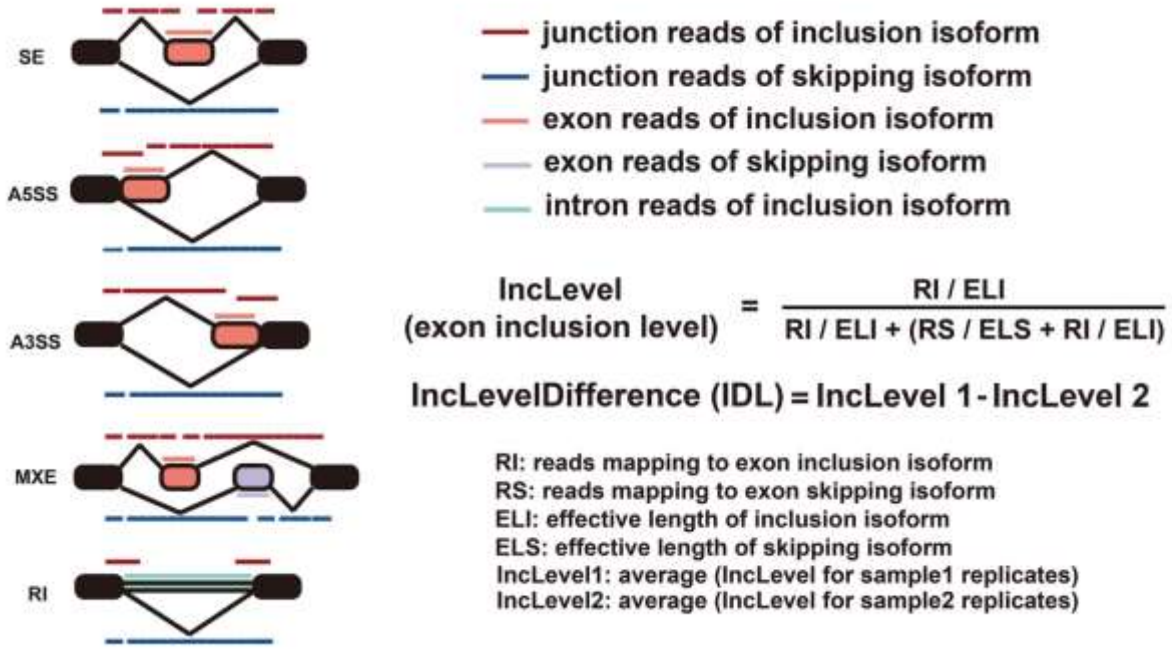

Figure S1. Schematic summary of alternative splicing categories and quantification principles. Alternative splicing can be divided into five main types: skipped exon (SE), alternative 5' splice site (A5SS), alternative 3’ splice site (A3SS), mutually exclusive exon (MXE), and retained intron (RI). The horizontal line represents different types of reads. The formula demonstrates how to calculate the exon inclusion level (IncLevel) in rMATS using read mapping to junctions or exons (or introns) of different isoforms. The IncLevelDifference (IDL) is the difference between the IncLevel of the previous sample (IncLevel 1) and the IncLevel of the subsequent sample (IncLevel 2), ranging from -1 to 1.

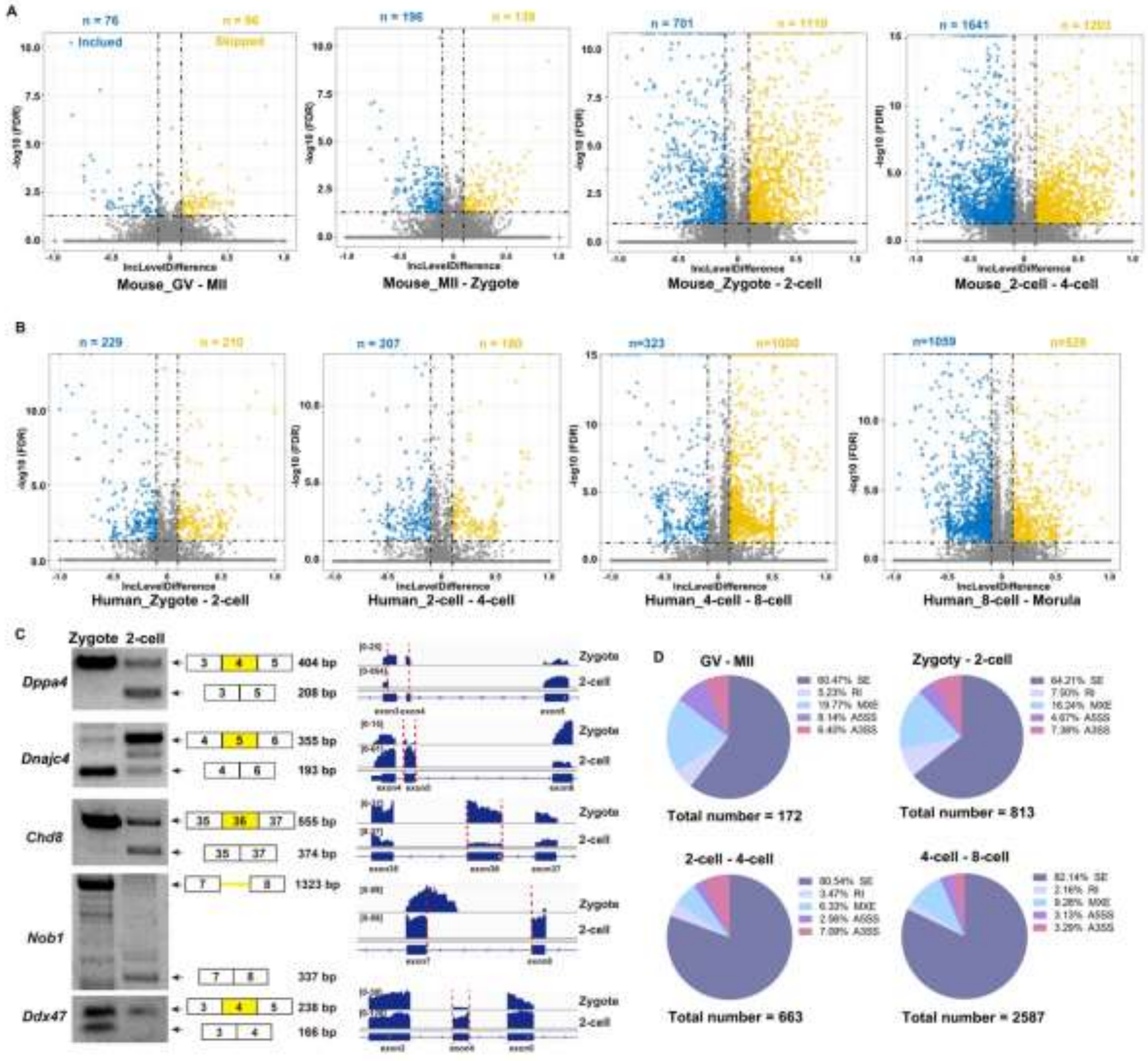

Figure S2. Dynamic pattern of alternative splicing during the development of oocytes and preimplantation embryos in mouse and human. A: The alternative splicing events (ASEs) of different stages from mouse germinal vesicle (GV) oocytes and 4-cell embryos are shown with a volcano map. **B:** The ASEs of different stages from human zygotes and morula embryos are shown with a volcano map. **C:** The verification of different isoforms in 2-cell mouse embryos by reverse transcription-polymerase chain reaction (RT-PCR), and the visualization results from the Integrative Genomics Viewer. **D:** The ratio of different ASEs at different stages of mouse and human embryos.

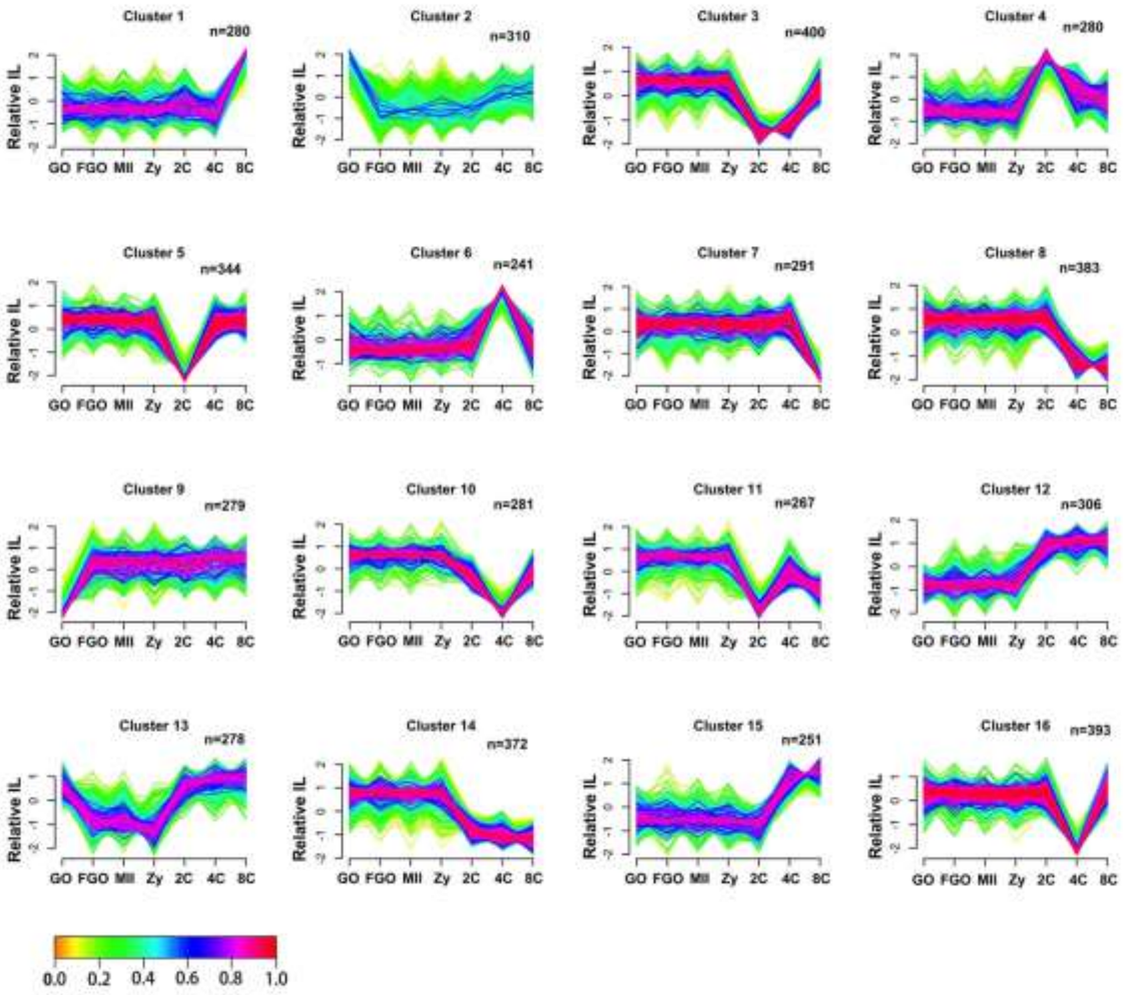

Figure S3. Different clusters of exon IncLevel from Mfuzz in mouse. Profiles of scaled IncLevels (ILs) for each exon within each Mfuzz cluster in mouse. The color of each profile line corresponds to the “Mfuzz membership score” of the exon, i.e. how similar it is to the profile of the median values (from orange-yellow [low similarity] to purple-red [high similarity]). The number of events (n) is indicated for each cluster.

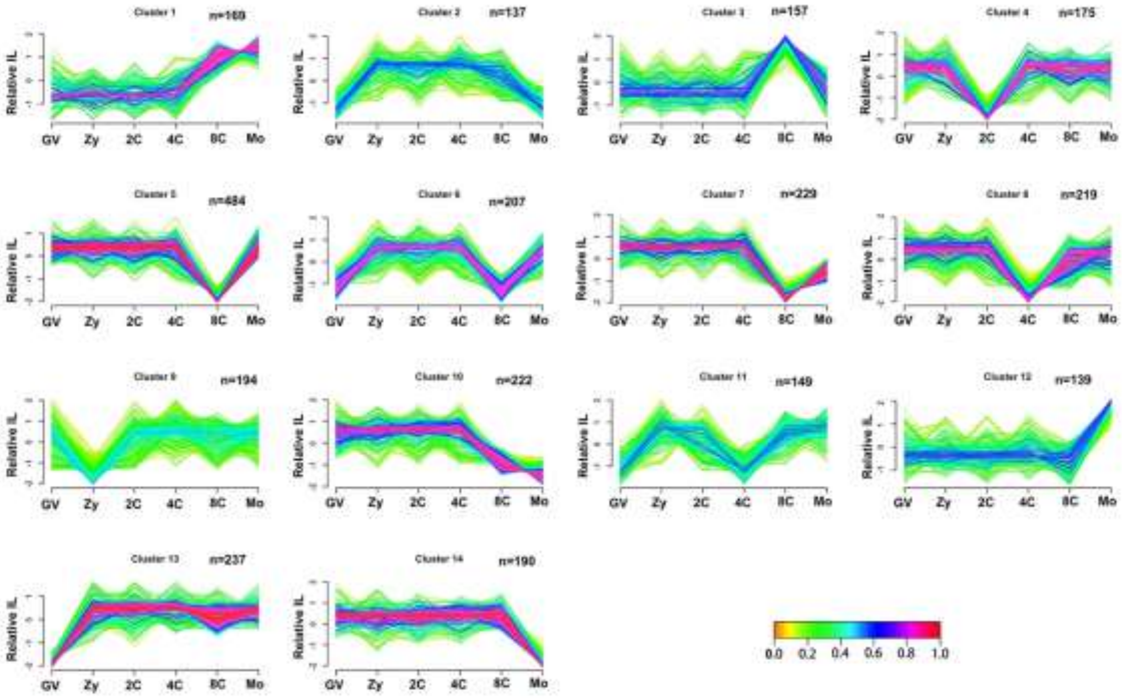

Figure S4. Different clusters of exon IncLevel from Mfuzz in human. Profiles of scaled IncLevels (ILs) for each exon within each Mfuzz cluster in human. The color of each profile line corresponds to the "Mfuzz membership score" of the exon, i.e. how similar it is to the profile of the median values (from orange-yellow [low similarity] to purple-red [high similarity]). The number of events (n) is indicated for each cluster.

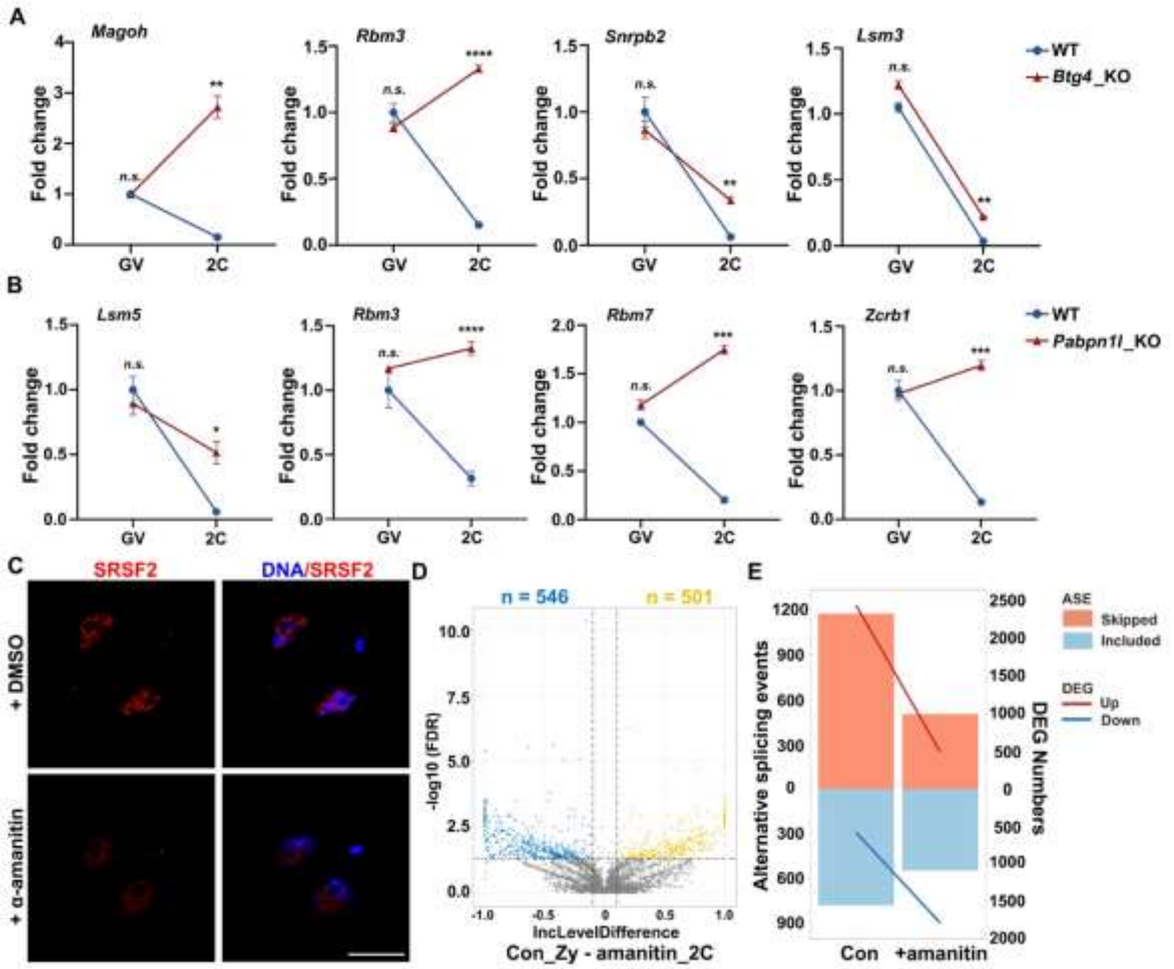

Figure S5. Failure of splicing factor decay/activation or transcription leads to zygotic splicing activation failure. A: RT-qPCR results showing the expression levels of splicing factors in *Btg4*- knockout (KO) mouse GV oocytes and 2-cell embryos. **B:** RT-qPCR results showing the expression levels of splicing factors in *Pabpn1l*-KO mouse GV oocytes and 2-cell embryos. Error bars, standard error of the mean (SEM). $^{*}P < 0.05$, $^{**}P < 0.01$, $^{***}P < 0.001$. **C:** Immunofluorescent staining showing the speckles of SRSF2 and DAPI in 2-cell embryos after treatment with α-Amanitin (100 nM) or DMSO. Scale bar, 20 μm. **D:** Volcano plot showing changes in transcript isoforms skipped or included in control zygotes and 2-cell embryos treated with α-Amanitin. **E:** ASEs (FDR < 0.05 and |ILD| > 0.1) and differentially expressed genes (DEGs, FDR < 0.05 and FPKM |Log2 fold change| > 2 or < 0.5) number. FDR, false discovery rate; ILD, IncLevelDifference; FPKM, fragments per kilobase of transcript per million mapped reads. Con group: control zygotes and control 2-cell embryos. + α-Amanitin group: control group zygotes and 2-cell embryos treated with α-Amanitin.

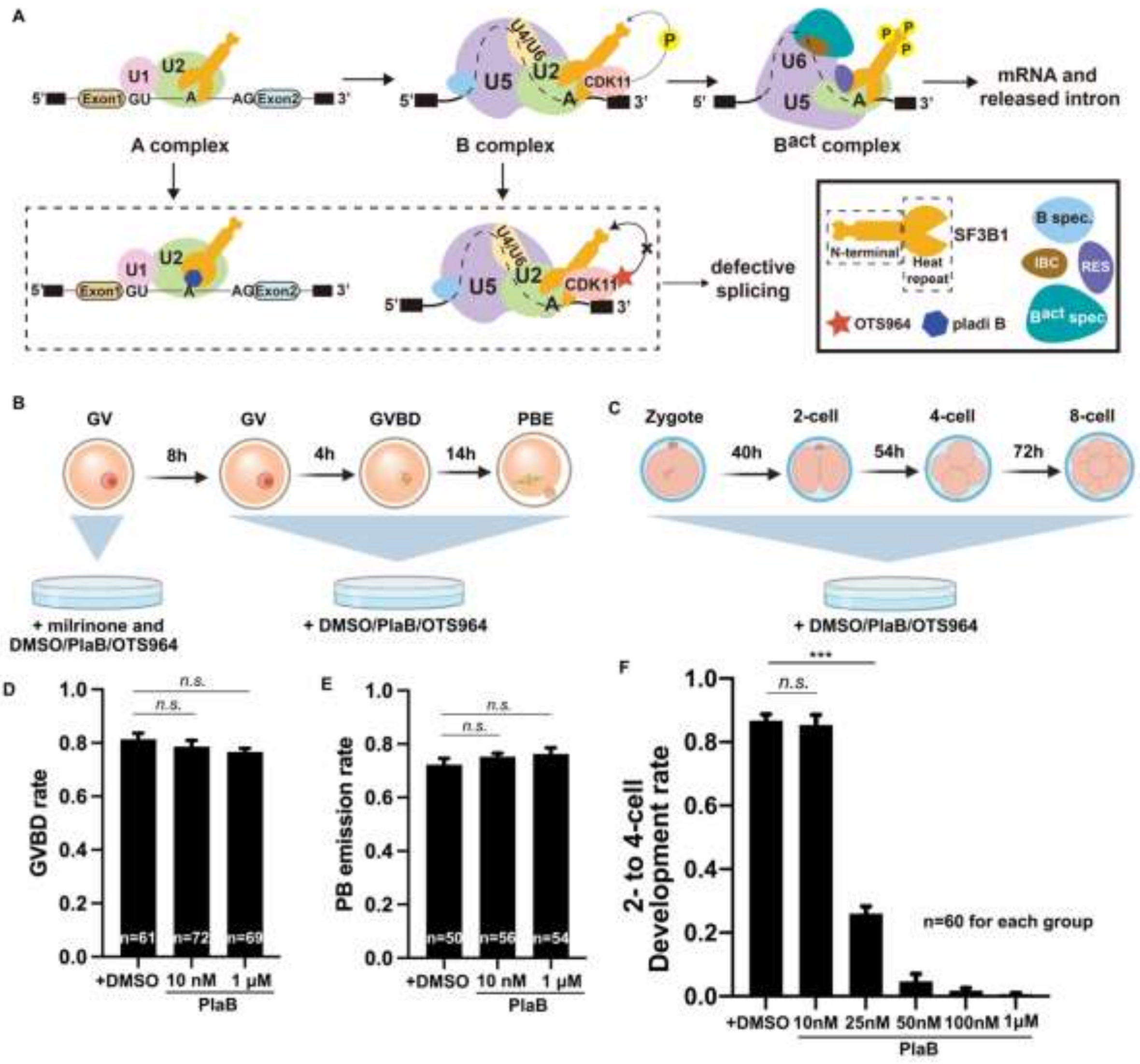

Figure S6. Effects of PlaB and OTS964 on alternative splicing and embryonic development. A: The working model of the effects of pladienolide B (PlaB) and OTS964 on SF3B1. **B and C:** Schematic diagram of oocytes and embryo cultures *in vitro*. All oocytes and embryos were collected *in vivo*. **D:** Comparison of germinal vesicle breakdown (GVBD) rates in cultured oocytes treated with dimethyl sulfoxide (DMSO) or PlaB. **E:** The rates of polar body emission (PBE) in cultured oocytes treated with DMSO or PlaB. When oocytes had undergone GVBD within 6 h, they were selected for further culture. **F:** The development rates of 2-cell to 4-cell embryos after treatment with DMSO or PlaB. The numbers of analyzed embryos are indicated (n). n = 3 biological replicates. Error bars, SEM; n.s.: non-significant. ***$P < 0.001$ by two-tailed Student's t-test.

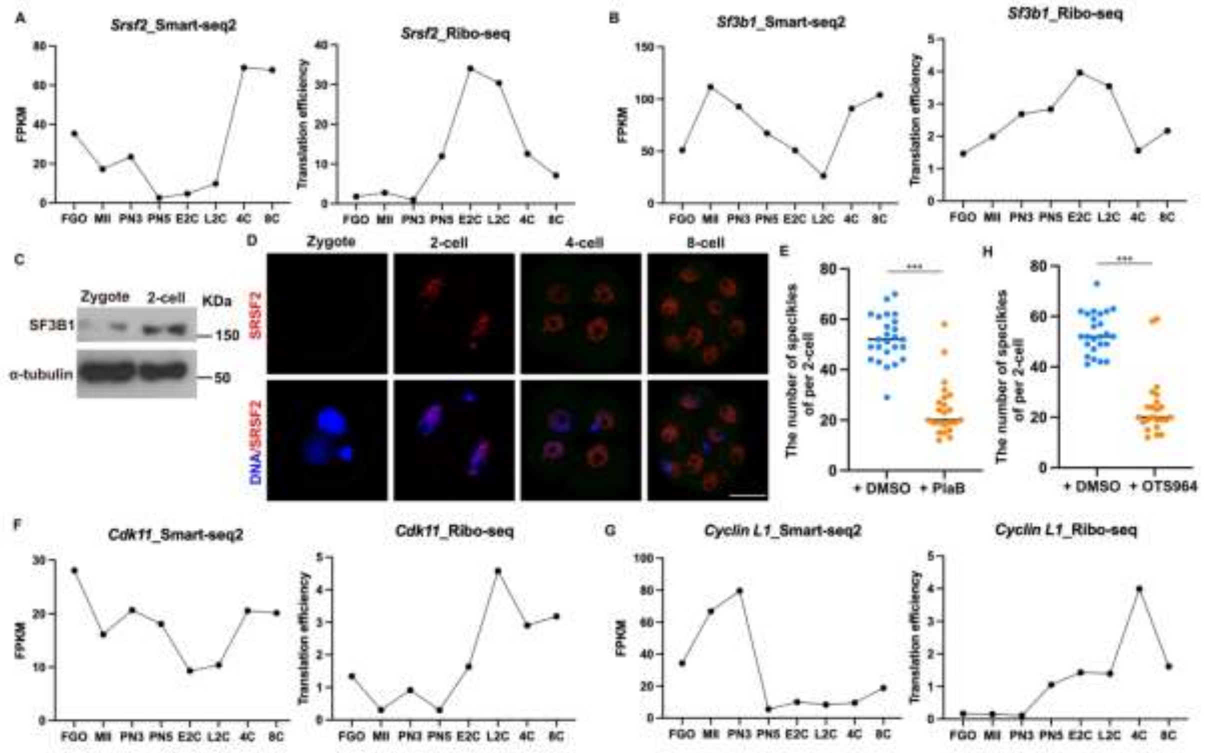

Figure S7. Expression patterns of SRSF2, SF3B1, CDK11, and Cyclin L1 in oocytes and early embryos. A and B: The expression pattern of *Srsf2* and *Sf3b1* during the maternal-to-zygotic transition (MZT) is derived from Smart-seq2 and Ribo-seq data. Translation efficiency analysis was calculated by the ratio of Ribo-seq and Smart-seq2 (FPKM + 1/FPKM + 1). **C:** Western blotting results of SF3B1 in Zygote and 2-cell embryos. **D:** Immunofluorescent staining showing the speckles of SRSF2 and DAPI in Zygote, 2-cell, 4-cell, and 8-cell stages. Scale bar, 20 μm. **E:** Quantification of the number of SRSF2 speckles per 2-cell embryo in Fig 3D. **F and G:** The expression pattern of *Cdk11* and *Cyclin L1* during the maternal-to-zygotic transition (MZT) is derived from Smart-seq2 and Ribo-seq data. Translation efficiency analysis was calculated by the ratio of Ribo-seq and Smart-seq2 (FPKM + 1/FPKM + 1). **H:** Quantification of the number of SRSF2 speckles per 2-cell embryo in Fig 4E.

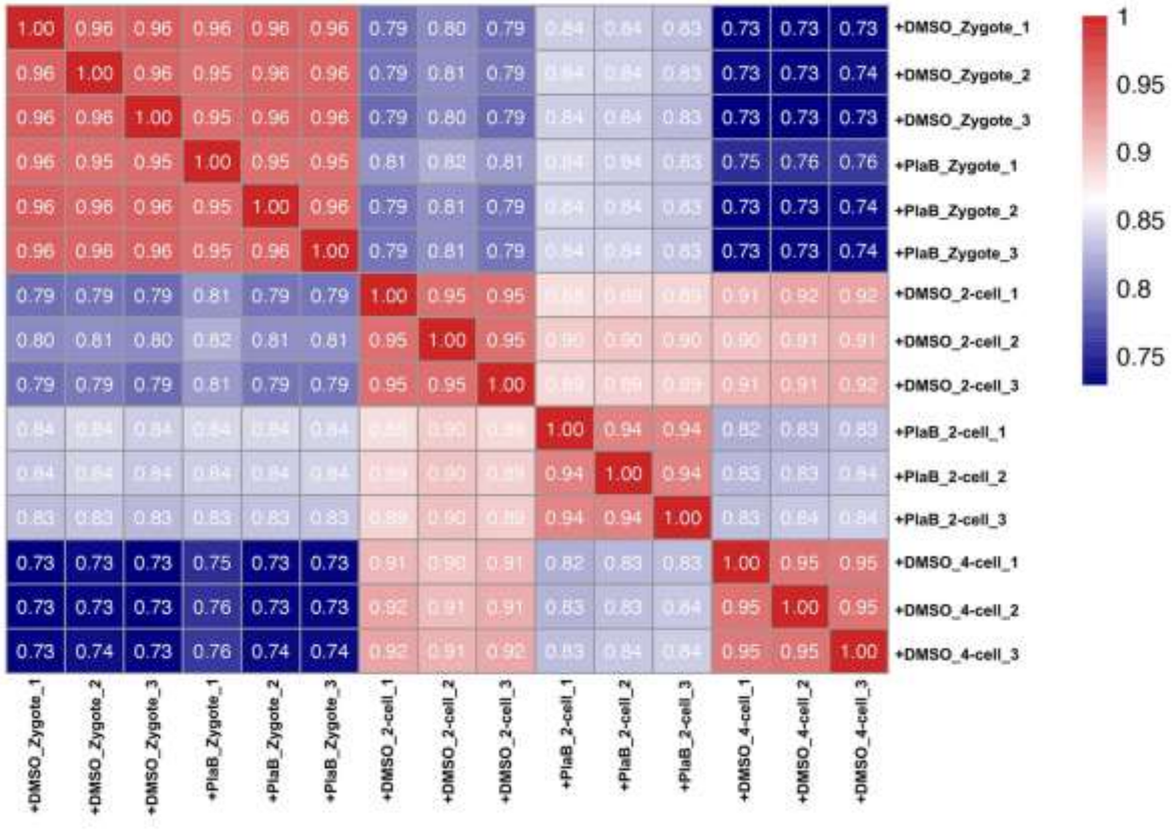

Figure S8. Analyses of the correlations between the indicated biological replicates of zygotes, 2-cell embryos, and 4-cell embryos treated with DMSO or PlaB (100 nM). The numbers correspond to R^2 (Pearson's correlation coefficient) values between the indicated replicates and the correlation strength is indicated by the color code.

Supplementary Tables

Table S1. Mfuzz cluster information for all skipped exon events in mouse oocytes and early embryos (in a separate Excel file)

Table S2. Mfuzz cluster information for all skipped exon events in human oocytes and early embryos (in a separate Excel file)

Table S3. Antibody information

**Protein name**	**Manufacturer (catalogue number)**	**Applications (working dilution)**	**Website Link***
**SRSF2**	Sigma-Aldrich (SAB4200725)	IF (1:100)	https://www.sigmaaldrich.cn/CN/zh/product/sigma/sab4200725
**SF3B1**	ABclonal (A15801)	IF (1:200)	https://abclonal.com.cn/catalog/A15801
**p-T313-SF3B1**	Affinity (AF2401)	IF (1:200)<br>WB (1:1000)	https://www.affbiotech.com/goods-15700-AF2401-Phospho_SF3B1_Thr313_Antibody.html
**MuERVL-Gag**	Huabio (ER50102)	IF (1:200)	https://www.huabio.com/products/muervl-gag-antibody-polyclonal-er50102
**GAPDH**	Trans (HC301)	WB (1:1000)	https://www.transgenbiotech.com/loading_controls/proteinfind_anti_gapdh_mouse_monoclonal_antibody.html
**RNA PolII (pS2)**	Abcam (ab5095)	IF (1:40000)	http://www.abcam.cn/rna-polymerase-ii-ctd-repeat-ysptsps-phospho-s2-antibody-chip-grade-ab5095.html
**α-Tubulin**	Sigma (F2168)	WB (1:1000)	https://www.sigmaaldrich.cn/CN/zh/product/sigma/f2168
**FLAG**	Sigma-Aldrich (F3165)	WB (1:2000)	http://www.sigmaaldrich.com/catalog/product/sigma/f3165?lang=zh&region=CN
**DDB1**	Epitomics	WB (1:2500)	http://www.epitomics.com/products/search/DD

	(3821-1)		B1

Table S4. Primer sequences

**Primer name**	**Genes targeted**	**Application**	**Sequences (5′-3′)**
Dppa4-F	*Dppa4*	Reverse transcription PCR	5′-ACAAGACACTGAGACGCCAGGACAG-3′
Dppa4-R			5′-GTGCTGACTTCCTCATAGAGCATAGGAGG-3′
Dnajc4-F	*Dnajc4*	Reverse transcription PCR	5′-GGAGCTGAATGAGGCATATCGAG-3′
Dnajc4-R			5′-GGCTGTAATGATCCGGTCCT-3′
Idh3g-F	*Idh3g*	Reverse transcription PCR	5′-CAACCATAACCTGCCACCGT-3′
Idh3g-R			5′-GCACGGCCGTCACTTTCTTG-3′
Chd8-F	*Chd8*	Reverse transcription PCR	5′-AGTTTACGAAACTTCGGCGAGGC-3′
Chd8-R			5′-TTGTCCTACGGACCCGTTTCTTGC-3′
Chd8-R	*Nob1*	Reverse transcription PCR	5′-TTGTCCTACGGACCCGTTTCTTGC-3′
Nob1-R			5′-GTGGGTTCAGAACTTTCG-3′
Ddx47-F	*Ddx47*	Reverse transcription PCR	5′-TTGGCCTTTCAGATCTCTGAGC-3′
Ddx47-R			5′- CCATGTTCAGTATCCGGTCTGC-3′
Ube2a-F	*Ube2a*	Real-time PCR	5′-ATGAGGGACTTCAAGAGGTTACA-3′
Ube2a-R			5′-TCTGCATAGACGTTAGGATGGA-3′
Lsm4-F	*Lsm4*	Real-time PCR	5′-CAGCGCAGAATCACCCAT-3′
Lsm4-R			5′-CGCACCATGTCAATGATCTCA-3′
Clcc1-F	*Clcc1*	Real-time PCR	5′-TCCTTTGTGAATGTCTGTTGCT-3′
Clcc1-R			5′-CTGAGATTTCCTCATCGTTCCTG-3′
Cct2-F	*Cct2*	Real-time PCR	5′-CTTTCCCTCGCACCTGTTAAT-3′
Cct2-R			5′-TCTGCATAGACGTTAGGATGGA-3′
Dppa5a-F	*Dppa5a*	Real-time PCR	5′-ATGATGGTGACCCTCGTGAC-3′
Dppa5a-R			5′- ACCTCGATAAGTTCTTCGGGAG-3′
H2afz-F	*H2afz*	Real-time PCR	5′-CCAAGACAAAGGCGGTTTCC-3′
H2afz-R			5′- TCCTGCCAACTCAAGTACCTC-3′

Utf1-F	*Utf1*	Real-time PCR	5′-TGTCCCGGTGACTACGTCT-3′
Utf1-R			5′- CCCAGAAGTAGCTCCGTCTCT-3′
Upp1-F	*Upp1*	Real-time PCR	5′-ACAGGAACTGAAGCAAAGGAC-3′
Upp1-R			5′- GTTGAAATGGTAGAGCACGTCTT-3′
Magoh-F	*Magoh*	Real-time PCR	5′-ACTTTTACCTGCGTTACTACGTG-3′
Magoh-R			5′- GTTGTTGGCGTATCGCAATTT-3′
Lsm5-F	*Lsm5*	Real-time PCR	5′-ACTCCTGCCACTAGAGCTTGT-3′
Lsm5-R			5′- CTCCGGGAACCAGCATTGTTA-3′
Rbm3-F	*Rbm3*	Real-time PCR	5′-TCCCTCCCAGATAGGTGTAATCC-3′
Rbm3-R			5′- TCCTTTCTGTTCCTCTCAAGGT-3′
Zcrb1-F	*Zcrb1*	Real-time PCR	5′-TGATAAAGGCGAGCATCGCTA-3′
Zcrb1-R			5′- AGCATGTTTTTAGGACAGGCAT-3′
Rbm7-F	*Rbm7*	Real-time PCR	5′-GTGGGTAACCTGGAGACGAAG-3′
Rbm7-R			5′- TCATGTTTGAAGTTCACGAATGC-3′
Snrpb2-F	*Snrpb2*	Real-time PCR	5′-AAGAGATCCCTGTATGCCCTT-3′
Snrpb2-R			5′- GTGGATGAACCCAGTTCCTTAAA-3′
Lsm3-F	*Lsm3*	Real-time PCR	5′-ATGGCGGACGACGTAGATCA-3′
Lsm3-R			5′- AGCTCTCGGTCATTTCTCATTTT-3′

Table S5. Maternal and zygotic splicing factors defined by RNA-seq datasets from *Btg4*- and *Pabpn1l*-knockout mice (in a separate Excel file)
